# Molecular Evolution of Infectious Pancreatic Necrosis Virus in China

**DOI:** 10.3390/v13030488

**Published:** 2021-03-16

**Authors:** Kaiyue Duan, Jingzhuang Zhao, Guangming Ren, Yizhi Shao, Tongyan Lu, Lipu Xu, Xin Tang, Wenwen Zhao, Liming Xu

**Affiliations:** 1 Key Laboratory of Aquatic Animal Diseases and Immune Technology of Heilongjiang Province, Department of Aquatic Animal Diseases and Control, Heilongjiang River Fisheries Research Institute, Chinese Academy of Fishery Sciences, Harbin 150070, China; duankaiyue710@163.com (K.D.); zhaojingzhuang@hrfri.ac.cn (J.Z.); renguangming@hrfri.ac.cn (G.R.); shaoyizhi@hrfri.ac.cn (Y.S.); lutongyan@hrfri.ac.cn (T.L.); pro_tangxin@163.com (X.T.); ZWW19818928328@163.com (W.Z.); 2 Fish Disease Department of Beijing Fisheries Technical Extension Station, Beijing 100176, China; xulipu001@163.com; 3 Key Laboratory of Aquatic Animal Immune Technology, Key Laboratory of Fishery Drug Development, Pearl River Fisheries Research Institute, Chinese Academy of Fishery Sciences, Ministry of Agriculture and Rural Affairs, Guangzhou 510380, China

**Keywords:** rainbow trout, aquabirnavirus, infectious pancreatic necrosis virus, molecular evolution, genotype, divergence time

## Abstract

Passive virus surveillance was performed in twenty-nine salmon and trout farms from seven provinces and districts in China during the period 2017–2020. A total of 25 infectious pancreatic necrosis virus (IPNV) isolates were obtained, mainly from rainbow trout (*Oncorhynchus mykiss*). The molecular evolution of these Chinese IPNV isolates and the previously reported Chinese IPNV strains ChRtm213 and WZ2016 was analyzed, based on their VP2 gene coding region sequences (CDS). All 27 Chinese IPNV isolates clustered within genogroups I and V, with 24 of the IPNV isolates belonging to genogroup I (including ChRtm213 and WZ2016), and only three isolates clustering in genogroup V. The Chinese genogroup I IPNV isolates lacked diversity, composing six haplotypes with 41 polymorphic sites, and the identity of nucleotide and amino acid sequences among the entire VP2 gene CDS from these isolates was 97.44%–100% and 98.19%–100%, respectively. Divergence time analyses revealed that the Chinese genogroup I IPNV isolates likely diverged from Japanese IPNV isolates in 1985 (95% highest posterior density (HPD), 1965–1997), and diverged again in 2006 (95% HPD, 1996–2013) in China. Each of the three Chinese genogroup V IPNV isolates has a unique VP2 gene CDS, with a total of 21 polymorphic sites; the identity of nucleotide and amino acid sequences among all VP2 gene CDS from these isolates was 98.5%–99.5% and 98.6%–99.0%, respectively. The data demonstrate that genogroups I and V are more likely the currently prevalent Chinese IPNV genotypes.

## 1. Introduction

Infectious pancreatic necrosis (IPN), a severe viral aquatic disease, was first recorded in salmonids in the Canada in the 1941 [1]. To date, IPN has been found in many countries, such as France [2], Croatia [3], Turkey [4], Chile [5], Mexico [6], Finland [7], Norway [8], Spain [9], Japan [10], Iran [11], Korea [12], Poland [13], and so on, all of which have caused large economic losses in the salmonid culture industry. IPN virus (IPNV), which belongs to the genus *Aquabirnavirus*, is an unenveloped virus of about 60–65 nm in diameter [14,15] that forms a crystalline arrangement in host cells [16]. The IPNV genome is composed of two linear double-stranded RNA segments (segment A and segment B). Segment A contains two open reading frames (ORFs), which encode a polyprotein composed of VP2, VP4, and VP3 [17] and the non-structural protein VP5, respectively. Segment B encodes the RNA polymerase VP1 [18,19]. Aquabirnavirus can be divided into seven genotypes based on the VP2 gene; these include six genotypes (genogroups I–VI) of IPNV [2] and one genotype (genogroup VII) comprising Japanese aquabirnavirus [10]. A *Paralichthys olivaceu* birnavirus (POBV) was isolated from flounder in China in 2008 [20], and which belonged to the genogroup VII. Ten Aquabirnavirus serotypes have been identified: nine serotypes from serogroup A (A1–A9) and one serotype from serogroup B (B1).

IPNV was first identified from a rainbow trout (*Oncorhynchus mykiss*) farm in Shanxi province of China in the 1980s [21] and was subsequently reported by other provinces [22,23,24,25], caused significant economic losses to the rainbow trout industry in China. Although decades have passed since the first outbreak of IPN in China, no gene sequences of Chinese IPNV strains were available until the publication of the VP2 gene sequences of Chinese IPNV strains ChRtm213 [23] and WZ2016 [24], which were isolated in 2013 and 2016, respectively. Little is known about the prevalent genotypes, diversity, and evolution situation of IPNV in China. To address these questions, 25 IPNV strains isolated from nine fish farms across seven regions in China during the period from 2017–2020, along with the two previously reported Chinese IPNV isolates, were comprehensively analyzed here to reveal the phylogenetic evolution of IPNV in China.

## 2. Materials and Methods

### 2.1. Sample Collection and Virus Isolation

**Figure 1:**
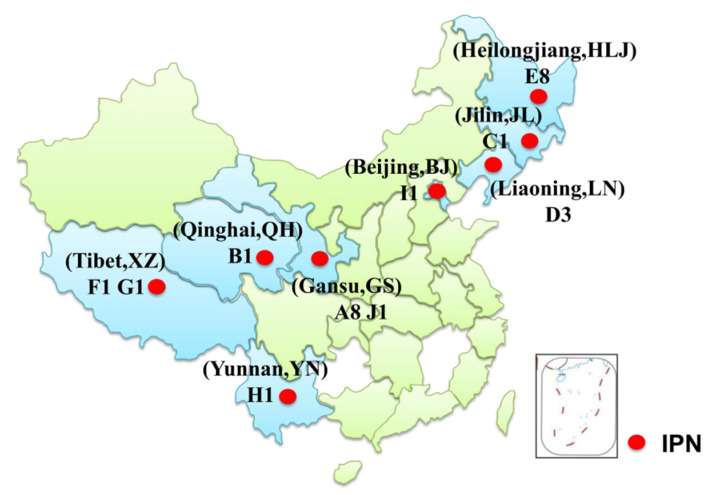
Map of the geographical locations where fish samples were collected and tested in our laboratory during the period 2013–2020. The sampling provinces and districts are marked in light blue. IPNV positive farm sites are indicated as random letters followed by the number of IPNV strains isolated from the farm within the corresponding province or district.

Passive surveillance of salmonid virus was performed, and total of 108 samples from 29 fish farms in seven Chinese provinces or district were tested during the period from 2017–2020 (Table 1). The provinces and districts are indicated in Figure 1. Diseased fish were collected by fish farmers or local fisheries management departments and sent on ice to the Department of Aquatic Animal Diseases and Control, Heilongjiang River Fisheries Research Institute, Chinese Academy of Fishery Sciences, Harbin, China. Samples were tested within 48 h of arrival, as in previous studies [26,27]. The liver, spleen, kidney, and head kidney of fish samples were pooled and centrifuged at 10,000 *g* for 5 min at 4 °C. After filtration, the supernatant of the pooled tissues was inoculated to monolayer cells and incubated at 15 °C for virus isolation. IPNV and infectious hematopoietic necrosis virus (IHNV) were chosen as target viruses for this study because both viruses have been commonly recorded in fish in China. The cell lines used for isolation of IPNV and IHNV were Chinook salmon embryo cells (CHSE-214) and epithelioma papulosum cyprini (EPC), respectively. The primers for IPNV detection (584 bp) were 5′ CAAGGCAACCGCAACYTACT 3′ (forward primer) and 5′ ATKGCAGCTGTGCACCTCAT 3′) (reverse primer), and the primers for IHNV detection (825 bp) were 5′ CATAGAAATAAAACAAGAGAGACTC 3′ (forward primer) and 5′ CCTCGTATTGTGTTTCGGAAATCT 3′) (reverse primer). The one-step reverse transcription polymerase chain reaction (RT-PCR) for detection of IPNV and IHNV was performed by using PrimeScript™ One Step RT-PCR Kit Ver.2 (RR057A, TaKaRa, Japan). The one-step RT-PCR reaction system included 2.5 μL RNA (50 ng/μL), 1 μL mixture of forward and reverse primer (10 pmol/μL), 12.5 μL PrimeScript 1 step buffer, 1 μL PrimeScript 1 step enzyme, 3 μL H_2_O. Procedures of the one-step RT-PCR were as follows: reverse transcription at 50 °C for 30 min, predenaturation at 95 °C for 4 min, denaturation at 95 °C for 30 s, annealing at 55 °C for 30 s, extension at 72 °C for 60 s with 27 cycles, and final extension at 72 °C for 10 min. Each RT-PCR product was analyzed by 1% agarose gel electrophoresis, and those with target band were sent to be sequenced by the Comate biological company (Changchun, China).

### 2.2. IPNV Isolates Selected for Sequence Analysis

A total of 27 IPNV isolates were selected for analysis in this study; 25 of them were isolated in the passive surveillance conducted during 2017–2020, and the other two isolates were ChRtm213 [23] and WZ2016 [24]. ChRtm213 was isolated by our laboratory from Yunnan province in 2013 (Figure 1), and WZ2016 was isolated by other scholars from Sichuan province. The 25 IPNV strains were named according to the province or district from which they were isolated and their isolation year. For example, an IPNV strain that was isolated from Heilongjiang province in 2019 would be named as HLJ2019, followed by an Arabic numeral that indicates the order of isolation. Regarding the 25 IPNV isolates, eight were isolated from Heilongjiang province, three were isolated from Liaoning province, nine were isolated from Gansu province, two were isolated from Tibet, and one was isolated from each of the Jilin and Qinghai provinces and Beijing.

### 2.3. Amplification of the IPNV VP2 Gene Coding Sequence

After two passages of IPNV isolates in CHSE-214, virus RNA was extracted from cell culture supernatant by using TRIzol reagent and used to conduct one-step RT-PCR to amplify the VP2 gene coding sequence (CDS). Primers for amplification of the VP2 gene CDS of genogroup I IPNV (1326 nt) (F1: 5′GCCCTTTCTAACAAACAACC’3, R2: 5′ GAGCCGCCATTGGGAAGA’3) were designed according to the genome of the IPNV ChRtm213 (accession number: KX234591) [23] and primers for amplification of pVP2 gene CDS of genogroup V IPNV (1536 bp) were as used in a previous study [15]. The one-step RT-PCR was performed by using PrimeScript™ One Step RT-PCR Kit Ver.2 The one-step RT-PCR reaction system included 2.5 μL RNA (50 ng/μL), 1 μL mixture of forward and reverse primer (10 pmol/μL), 12.5 μL PrimeScript 1 step buffer, 1 μL PrimeScript 1 step enzyme, 3 μL H_2_O. Procedures of the one-step RT-PCR were as follows: reverse transcription at 50 °C for 30 min, predenaturation at 95 °C for 4 min, denaturation at 95 °C for 30 s, annealing at 55 °C for 30 s, and extension at 72 °C for 120 s with 27 cycles, the final extension was 10 min at 72 °C. All virus samples were tripled amplified by one step RT-PCR, and each RT-PCR product was analyzed by 1% agarose gel electrophoresis then sequenced by the Comate biological company (Changchun, China).

### 2.4. Sequence Alignment and Analysis

The raw VP2 gene sequences of IPNV isolates were processed multiple alignment in DNAman software (version 8.0, Lynnon Biosoft, Quebec, Canada) to obtain the VP2 gene CDS. In alignment progress, the full alignment method was used. In Pairwise Alignment step, the value of Gap Penalty was 7, the no. of Top was 5, K-tuple was 3, and window size was 5. In the multiple alignment steps, the value of Gap Open Penalty was 10, Delay Divergent Seqs was 30%, and Gap Extension was 5. Alignments were checked manually to confirm that no artificial gaps were introduced. The amino acid sequence was translated using the Expert Protein Analysis System World Wide Web server (ExPASy, http://www.expasy.org (accessed on 3 February 2021)). Nucleotide and amino acid sequence identity of Chinese IPNV with other reference birnavirus strains (genogroups I–VI) were calculated using MegAlign software. The analyses of the variable polymorphic site, haplotype diversity, and nucleotide diversity were performed by using DnaSP v5 [28].

### 2.5. Phylogenetic Analysis

The phylogenetic tree for the Chinese IPNV strains was constructed based on the VP2 gene CDS using Bayesian Inference method with MrBayes in PhyloSuite software [29]. The independent Markov Chain Monte Carlo (MCMC) analyses were run for 2.0 × 10^6^ generations, with samples drawn every 200 generations. The best nucleotide substitution model GTR + F + I + G4 was chosen by ModelFinder in PhyloSuite software, according to the Bayesian Information Criterion standard. To ensure all parameters were converged, the Tracer 1.7 software (University of Edinburgh, Edinburgh, UK) [30] was used to check the effective sample size (ESS) of each parameter to make sure all ESS greater than 200 [30]. Reference aquabirnaviruses belonging to genogroups I–VII were contained in this study (Table 2).

### 2.6. Inference of Divergence Time

To assess whether the sequence data had a sufficient time signal to infer the divergence time and to calculate the evolution rate, we first performed a date randomization test (DRT) [31] on our IPNV sequence data, using the standard date randomization test method [30]. When sequence data had sufficient time signal, the sampling times of the viral isolates were used to calibrate the molecular clock by using the dated-tip method [32]. As the sufficient time signal was only found in our genogroup I IPNV sequence data (Supplementary Figure S1), we conducted a Bayesian phylogenetic analysis by using the Bayesian Evolutionary Analysis Sampling Tree (BEAST) 1.10.4 software (University of Edinburgh, Edinburgh, UK) [33] to infer the evolutionary rate and divergence time of the Chinese genogroup I IPNV isolates. The best substitution model was selected by using the ModelFinder implemented in PhyloSuite software [29], following the Bayesian Information Criterion. To find the best clock model (strict or relaxed clock) and tree prior (constant size, exponential growth, or Bayesian skyline coalescent), we computed marginal likelihoods with six different model combinations using the stepping-stone sampling and path sampling methods [34]. The independent Markov Chain Monte Carlo (MCMC) analyses were run for 4.0 × 10^7^ generations, with samples drawn every 40,000 generations. The convergence of all parameters was checked using Tracer 1.7 [35] to ensure the effective sample size (ESS) of each parameter was greater than 200 [35]. After discarding the first 10% of samples as burn-in, maximum clade credibility (MCC) phylogenetic trees were generated by using TreeAnnotator v1.10.4 software (University of Edinburgh, Edinburgh, UK) and were visualized with FigTree v1.4.3 software (University of Edinburgh, Edinburgh, UK).

### 2.7. Prediction of Virulence of Chinese Genogroup V IPNV Isolates

Previous studies found that the virulence of the genogroup V IPNV strains in Atlantic salmon (Salmo salar) is related to the amino acid at positions 217, 221, 247, 286, 288, and 500 of the VP2 protein [8,36,37,38,39], and comprehensive studies have been performed on positions 217 and 221 [39]. Highly virulent isolates contain threonine and alanine at positions 217 and 221, respectively, whereas moderate- and low-virulence strains contain proline and alanine at positions 217 and 221, respectively. Strains containing a threonine at position 221 (T221) of the VP2 amino acid sequence are almost avirulent, regardless of the residue at position 217 [39]. Based on the previous studies, the virulence of our three genogroup V IPNV isolates were predicted base on their VP2 protein sequence.

## 3. Results

### 3.1. Prevalence of IPNV in Chinese Fish Farms

A total of 108 samples from 29 fish farms in seven provinces and districts in China were tested during the period from 2017 to 2020 in our passive surveillance, and a total of twenty-five IPNV isolates were isolated from nine farms located across the seven provinces and districts (Table 1, Figure 1). Co-infection of IHNV and IPNV were observed from three rainbow trout samples from farm A in Gansu province and one rainbow trout sample from farm D (Table 3). All of the Chinese IPNV isolates were isolated mainly from triploid rainbow trout, ranging in size from 1.5 to 450 g. IPNV was also isolated from other fish species, including Hucho taimen, white-spotted char, brook trout, brown trout, masu salmon, and crucian carp (Table 3). The mainly clinical signs of the IPNV infected salmonids with mortality included darkening of skin, exophthalmia, ascites, distended abdomen, grey faces, and hemorrhage of pyloric caecum in some cases. Only darkening of skin, ascites, and distended abdomen were observed in IPNV infected salmonids that showed no obvious mortality. No typical clinical signs were observed in a dead crucian carp that was co-cultured with the brook trout (50 ± 10 g) in farm E.

The mortality rate of the IPNV infected fish in the passive surveillance varied widely, ranging from 0%–80% (Table 1). Mortality was observed in all but one of the IPNV positive farms (Table 3). Relatively high mortality rates were observed in infected juvenile brown trout (~60%) and brook trout (~40%), but the highest mortality was observed in rainbow trout (80%). Compared with single IPNV infection in rainbow trout, mortality was constantly high in the co-infection of IHNV and IPNV cases (65~80%) (Table 3).

### 3.2. Sequence Analysis of the VP2 Gene CDS

The VP2 gene CDS of the 22 genogroup I IPNV isolates analyzed here was 1326 bp long, and they began with the translation initiation codon ATG and ended up with GCA. Among the three genogroup V isolates, the pVP2 gene CDS of two isolates (GS2020-1 and BJ2020-1) were 1536 bp long, and another isolate (GS2020-2) was 1539 bp long; they began with the translation initiation codon ATG and ended up with ACG.

**Figure 2:**
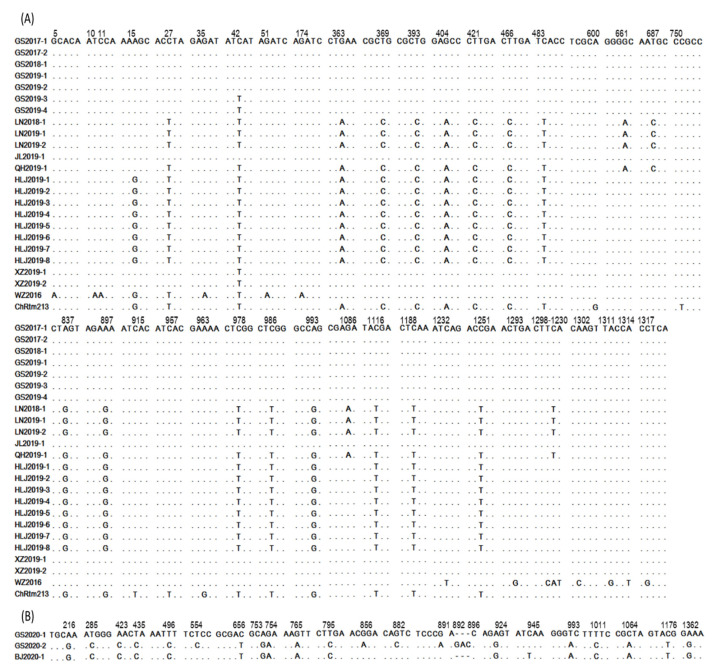
Distribution of polymorphic sites on the VP2 gene coding region sequences (CDS) of Chinese IPNV isolates collected from different farms during 2013 through 2020. (**A**) Chinese genogroup I IPNV isolates. GS2017-1 isolate was used as a reference strain here. (**B**) Chinese genogroup V IPNV isolates. GS2020-1 isolate was used as a reference strain here. The numbers above nucleotides represent the position that polymorphic sites were located, and all of the polymorphic sites were indicated by representing the corresponding nucleotides that differ from the reference isolates.

According to the results of the sequence identity comparison, 24 of the 27 analyzed Chinese IPNV strains, including ChRtm213 and WZ2016, had the closest relationship with genogroup I IPNV, with a nucleotide and amino acid identity of 90.2%–98.5% and 97.2%–99.4% (Table 4), respectively. This finding indicates that the majority of IPNV strains isolated in our surveillance likely belong to genogroup I. These 24 strains were divided into six haplotypes. Isolates from Gansu and Jilin provinces share the same haplotype, and those from Liaoning and Qinghai provinces share another haplotype. In addition, these strains contain a total of 41 polymorphic sites on the VP2 gene CDS (Figure 2) and the identity of nucleotide and amino acid sequences of the VP2 gene CDS from these Chinese genogroup I isolates is 97.44%–100% and 98.19%–100%, respectively.

Three of the 27 analyzed IPNV strains, isolated only from rainbow trout, were found to have the closest relationship with genogroup V IPNV, with a nucleotide and amino acid identity of 97.1%–100% and 97.6%–100%, respectively (Table 4). This finding indicates that these three IPNV strains probably belong to genogroup V. The three genogroup V isolates were divided into three haplotypes and contain a total of 21 polymorphic sites on the VP2 gene CDS. The identity of nucleotide and amino acid sequences on the VP2 gene CDS from these Chinese genogroup V IPNV isolates is 98.5%–99.5% and 98.6%–99.0%, respectively. Besides, three bases (GAC) were inserted between 892 (A) and 896 (C) in GS2020-2 compared with strains BJ2020-1 and GS2020-1. The distribution of polymorphic sites is shown in Figure 2.

### 3.3. Phylogenetic Analysis of Chinese IPNV

**Figure 3:**
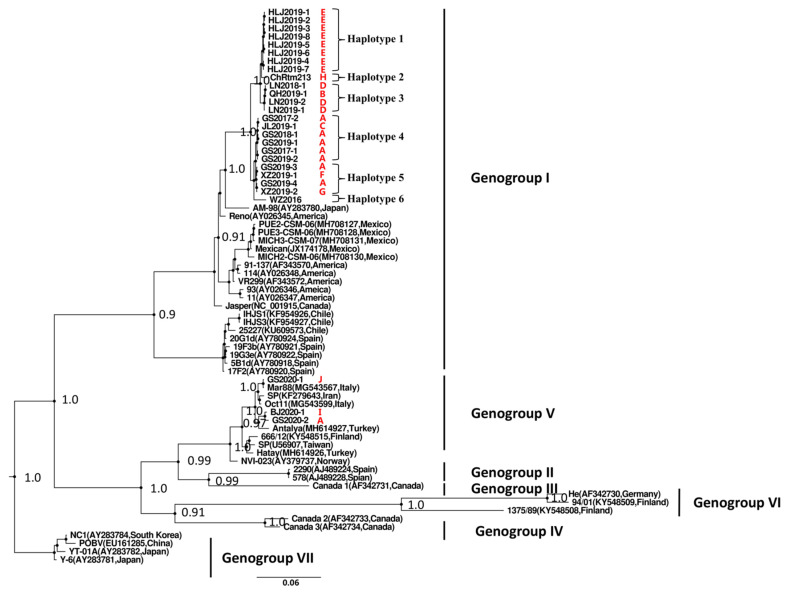
Phylogenetic tree of IPNV isolates constructed based on the VP2 gene CDS by using Bayesian Inference method. The main nodes with pp value of higher than 0.9 are labeled. All of the Chinese IPNV isolates (red labels) clustered within genogroup I and V, respectively. Red capital letters indicated fish farms. Information of IPNV isolates used here was listed in Table 1 and Table 3.

A phylogenetic analysis of all Chinese IPNV isolates from the present study along with reference strains from genogroups I–VII was conducted by using PhyloSuite software based on the VP2 gene CDS sequences, and the resulting phylogenetic tree is shown in Figure 3. The Chinese IPNV strains clustered within two genogroups, genogroup I and genogroup V. Among the analyzed strains, 24 strains clustered with the genogroup I IPNV isolates, and three strains clustered with the genogroup V IPNV strains. Six haplotypes were found in these Chinese genogroup I IPNV isolates and three haplotypes were found in these Chinese genogroup V IPNV isolates (Figure 3). The Chinese genogroup I IPNV isolates were found to have a closer relationship with Japanese IPNV strain AM-98 (posterior probability, 1.0). All of the Chinese genogroup I IPNV isolates clustered together and formed a phylogenetic monoclade within the genogroup I branch. The Chinese genogroup V IPNV isolates were found to have a closer relationship with Turkey strain Antalya (posterior probability, 0.97) and Italy strain Mar88 (posterior probability, 1.0) (Figure 3).

### 3.4. Inference of Divergence Time and Evolution Rate

**Figure 4:**
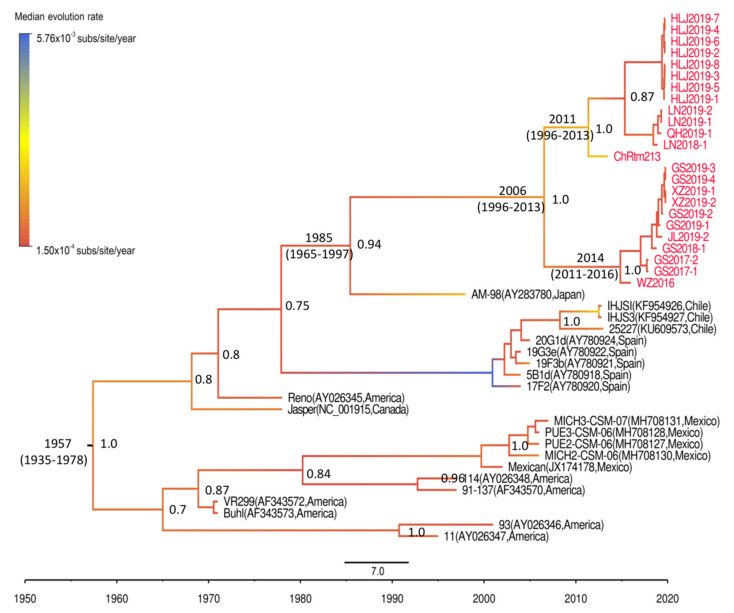
Maximum clade credibility (MCC) tree for genogroup I IPNV strains based on the VP2 gene CDS sequences, constructed using BEAST 1.10.4 software (University of Edinburgh, Edinburgh, UK). Posterior probability values are shown at each node, and branches with different color lines show the median evolution rate of each branch. The divergence time of each node, including the 95% highest posterior density, is shown only when the posterior probability value of the corresponding node is higher than 0.9.

Because the GTR + F + I + G4 substitution model and the combination of uncorrelated lognormal relaxed clock and Bayesian skyline coalescent tree prior produced the best fit for our data (Supplementary Tables S1 and S2), the Bayesian phylogenetic analysis of the Chinese genogroup I IPNV strains was conducted with these parameters. The results showed that the divergence time of genogroup I IPNV was 1957 (95% highest posterior density (HPD), 1935–1978). The divergence time between the Chinese and Japanese IPNV strains was estimated as 1985 (95% HPD, 1965–1997). In 2006 (95% HPD, 1996–2013), the Chinese IPNV strain evolved into two different branches: a branch including isolates from Yunnan, Heilongjiang, Liaoning, and Qinghai provinces, and a branch including isolates from Gansu, Jilin, and Sichuan provinces and Tibet district (Figure 4). The evolution rate of genogroup I IPNV was 1.12 × 10^−3^ subs/site/year (95% HPD, 6.5 × 10^−4^–1.6 × 10^−3^ subs/site/year).

### 3.5. Virulence Prediction of Chinese Genogroup V IPNV Isolates

The three Chinese genogroup V IPNV isolates had the same amino acid at each of the positions we checked on the VP2 protein sequence. There were Ile, Pro, Thr, Ala, Gly, Val, Tyr at positions of 199,217, 221, 247, 286, 288, 500 on the VP2 protein, respectively. The T221 signature was found in each of these three IPNV isolates (Table 5).

## 4. Discussion

As a highly contagious aquatic viral disease, IPN has caused serious economic losses to the salmon and trout industry worldwide [7]. The first outbreak of IPN in China occurred in Shanxi province in the 1980s [21], causing high mortality in juvenile rainbow trout on a large scale. Afterward, outbreaks of IPN were reported in Liaoning [25] and other province. IPN has since become one of the most important viral diseases hindering the salmon and trout industry in China. IPNV strains isolated during that early period were characterized by using neutralizing antibody assays as belonging to the Sp serotype [21]. However, phylogenetic analysis has not been performed on these Chinese IPNV isolates, and the genotypes of these previous Chinese IPNV isolates are still unknown. According to a previous study [41], all Sp serotype IPNV isolates belong to genogroup V, which suggests that the previous Chinese IPNV isolates likely to belong to genogroup V. Unfortunately, there is no available gene sequence to perform genotype analyses on the early isolated Chinese IPNV strains. Notably, the Chinese IPNV strains ChRtm213 [23] and WZ2016 [24], isolated in 2013 and 2016, respectively, belong to genogroup I not genogroup V, which raises many questions. How many genotypes of IPNV are circulating in China? What is the main prevalent genotype in China? Are genogroup V IPNV isolates still circulating in China? What is the relationship between the prevalent Chinese IPNV isolates? To address these questions, in 2017 we began an epidemiological investigation across twenty-nine rainbow trout farms located in seven provinces and districts. The investigation covered most of the rainbow trout culture regions in China. The annual rainbow trout product in these farms accounts for more than 50% of the annual rainbow trout production in China. During our epidemiological investigation, 25 IPNV strains were isolated; these were combined with the previously reported isolates ChRtm213 and WZ2016, yielding a total of 27 Chinese IPNV isolates analyzed in this study. We found that both genogroup I and genogroup V IPNV isolates exist in the Chinese fish farms and could even be found within a single farm, but most of the IPNV isolates belonged to genogroup I.

All of the Chinese genogroup I IPNV isolates had the highest similarity with Japanese IPNV strains, followed by the North American strains, which indicated that the genogroup I IPNV strains that are currently prevalent in China might share a recent common ancestor with strains from Japan. To investigate the transmission origin and divergence time of the Chinese IPNV strains, we inferred the divergence time of the Chinese IPNV isolates by conducting a BEAST phylogeny analysis. We found that genogroup I IPNV isolates began to diversify in the 1950s. This is consistent with the fact that the first outbreak of IPN in North America was recorded in the 1950s at farms cultivating freshwater brook trout [42]. The divergence time between the Chinese genogroup I IPNV and Japanese genogroup I IPNV strains was estimated to be the 1980s, which is consistent with the fact that an outbreak of IPN occurred in Chinese farms after the import of rainbow trout eggs from Japan in the 1980s [43]. These findings indicated that the Chinese genogroup I IPNV strains might have originated from Japan.

The Chinese genogroup V IPNV isolates had the highest similarity with IPNV strains from Turkey and Italy. However, there is no historical record showing that fish eggs or fry were introduced to China from Italy or Turkey. Regrettably, the sequence data from the Chinese genogroup V IPNV strains were lack of a sufficient time signal; we were unable to conduct the divergence time analyses. Although genogroup V IPNV was isolated in a Chinese fish farm after importation of trout eggs from Japan, the gene sequences of genogroup V IPNV strains of Japan are not currently available; we were unable to predict the likely origin of Chinese genogroup V IPNV isolates.

IPNV has a wide host range, such as rainbow trout [44] and Atlantic salmon [8]. It can also infect eels (*Anguilla anguilla*) [41] Atlantic cod (*Gadus morhua*) [45], Greenland halibut (*Sebastes mentella*) [45], deepwater redfish (S*alvelinus alpinus*) [45], Arctic char (*Scophthalmus maximus*) [45] and pike (*Esox lucius* L.) [46]. Because triploid rainbow trout is the main aquaculture species maintained in China, most of the fish samples tested in this study were from rainbow trout, and this species was the main host of the Chinese IPNV isolates. In addition to rainbow trout, genogroup I IPNV isolates were also isolated from other salmonids that shared the same water in farm E. Interestingly, genogroup I IPNV was also isolated from a dead crucian carp that was found within the IPNV-infected brook trout population in this farm. The other eight of the total nine fish farms tested in this study only raise rainbow trout, from which genogroup V IPNV was isolated. This might be the reason why genogroup V IPNV was only isolated from rainbow trout. The IPNV positive farms in this study were far away from each other, but transportation of fish fry in China happens frequently, which might be the reason that caused the spread of IPNV between fish farms in China.

Genogroup I and V IPNV strains have a wide distribution and have been comprehensively studied. Genogroup I IPNV strains have been identified in Chile, Spain, the United States, Canada, and Mexico, and genogroup V IPNV strains have been isolated in Norway, Finland, Scotland, Iran, Turkey, and Italy. Both two types of IPNV can cause mass death in trout juveniles. A previous study showed that genogroup V IPNV strains have a greater negative effect on rainbow trout compared with genogroup I IPNV strains [46]. However, Tapia, Barría [47] showed that genogroup I IPNV strains caused higher mortality in rainbow trout when they conducted artificial challenges. Comparison of mortality observed under the natural conditions in this study, we found that both genogroup I and genogroup V IPNV caused high mortality in rainbow trout. Due to the different sizes of hosts and environmental conditions, we cannot tell which genotype has higher mortality in rainbow trout. Based on a previous theory that was used to predict the virulence of genogroup V IPNV in Atlantic salmon, the Chinese genogroup V IPNV strains isolated in this study were predicted to be avirulent (Table 4). However, high mortality (50–80%) of rainbow trout was caused by the Chinese genogroup V IPNV strains under natural conditions, indicating the high virulence of these Chinese genogroup V IPNV in rainbow trout. A similar phenomenon was found in a previous study, in which IPNV strains that were predicted to be avirulent caused high mortality in rainbow trout [6]. As we know, the virulent and avirulent definitions connected to the different amino acid positions were mainly resulting from studies in Atlantic salmon. This may be different in the other species, particularly rainbow trout.

## 5. Conclusions

Both genogroup I and genogroup V IPNV isolates exist in the Chinese fish farms and could even be found within a single farm, but most of the IPNV isolates belonged to genogroup I. The two genotypes IPNV isolates caused high mortality of rainbow trout and other salmonids in China. The Chinese genogroup I IPNV isolates may have originated from Japan.

## Figures and Tables

**Table 1:** Passive surveillance for infectious pancreatic necrosis virus (IPNV) in Chinese salmonid fish from 2017 to 2020.

Province	# IPNV Positive Farms /# Farms Tested	# IPNV Positive Samples /# Samples Tested	Year(s) of IPNV Positive
Liaoning	1/15	3/36	2017–2019
Qinghai	1/1	1/6	2017, 2019
Jilin	1/5	1/5	2019
Heilongjiang	1/2	8/23	2017, 2019
Tibet	2/2	2/4	2019
Gansu	2/3	9/33	2017–2019, 2020
Beijing	1/1	1/1	2020
Total	9/29	25/108	2017–2019

# represents the numbers of fish farms or samples.

**Table 2:** Reference strains of aquabirnavirus used in this study.

Isolates	Genogroups	Host ^a^	Origin	Accession no.
Jasper	I	Trout	Canada	NC_001915
WZ2016	I	Rainbow trout	China	KX355401
Mexican	I	Rainbow trout	Mexico	JX174178
MICH2-CSM-06	I	Rainbow trout	Mexico	MH708130
MICH3-CSM-07	I	Rainbow trout	Mexico	MH708131
PUE2-CSM-06	I	Rainbow trout	Mexico	MH708127
PUE3-CSM-06	I	Rainbow trout	Mexico	MH708128
IHJSI	I	Rainbow trout	Chile	KF954926
IHJS3	I	Rainbow trout	Chile	KF954927
25227	I	Coho salmon	Chile	KU609573
114	I	Trout	America	AY026348
93	I	Trout	America	AY026346
AM-98	I	Amago salmon	Japan	AY283780
VR299	I	Trout	America	AF343572
Buhl	I	Trout	America	AF343573
Reno	I	Trout	America	AY026345
11	I	Trout	America	AY026347
91-137	I	Trout	America	AF343570
5B1d	I	Atlantic cod	Spain	AY780918
17F2	I	Greenland halibut	Spain	AY780920
19F3b	I	Greenland halibut	Spain	AY780921
20G1d	I	Deepwater redfish	Spain	AY780924
19G3e	I	Deepwater redfish	Spain	AY780922
578	II	Turbot	Spain	AJ489228
2290	II	Salmon	Spain	AJ489224
Canada 1	III	Trout	Canada	AF342731
Canada 2	IV	trout	Canada	AF342733
Canada 3	IV	Arctic char	Canada	AF342734
Mar88 ^b^	V	Rainbow trout	Italy	MG543567
Oct11 ^c^	V	Rainbow trout	Italy	MG543599
Antalya	V	Rainbow trout	Turkey	MH614927
Hatay	V	Rainbow trout	Turkey	MH614926
666/12	V	Rainbow trout	Finland	KY548515
NVI-023	V	Rainbow trout	Norway	AY379737
Sp	V	Trout	Taiwan	U56907
Sp	V	Rainbow trout	Iran	KF279643
1375/89	VI	Salmo trutta	Finland	KY548508
94/01	VI	Salmo salar	Finland	KY548509
He	VI	Pike	Germany	AF342730
Y-6	VII	Yellowtail	Japan	AY283781
YT-01A	VII	Yellowtail	Japan	AY283782
NC1	VII	Flounder	South Korea	AY283784
POBV	VII	Japanese flounder	China	EU161285

^a^ Trout: the specific species unknown; Latin names of hosts are as follows: rainbow trout, *Oncorhynchus mykiss*; Coho salmon, *Oncorhynchus kisutch*; Amago salmon, *Oncorhynchus masou ishikawae*; Atlantic cod, *Gadus morhua*; Greenland halibut, *Reinhardtius hippoglossoides*; deepwater redfish, *Sebastes mentella*; Arctic char, *Salvelinus alpinus*; turbot, *Scophthalmus maximus*; brown trout, *Salmo trutta*; Pike, *Esox lucius L*.; yellowtail, *Seriola quinqueradiata*; flounder, *Platichthys flesus*; Japanese flounder, *Paralichthys olivaceus*. ^b^ The full name of Italy strain Mar88 was IPNV/O. mykiss/I/PN/208/Mar88. ^c^ The full name of Italy strain Oct11 was IPNV/O. mykiss/I/TN/394/Oct11.

**Table 3:** The Chinese IPNV isolates used in this study.

Isolates	Provinces (Districts)	Accession No.	Farms ^a^	Date of Sampling	Host ^b^	Host Size (g)	Mortality (%) ^c^
GS2017-1 *	Gansu	MW662084	A	2017.10	Triploid rainbow trout	45 ± 5	65
GS2017-2 *	Gansu	MW662085	A	2017.10	Triploid rainbow trout	4 ± 1	75
GS2018-1 *	Gansu	MW662086	A	2018.08	Triploid rainbow trout	20 ± 2	80
GS2019-1	Gansu	MW662087	A	2019.02	Triploid rainbow trout	1.5 ± 0.5	50
GS2019-2 *	Gansu	MW662088	A	2019.05	Triploid rainbow trout	3 ± 1	78
GS2019-3	Gansu	MW662089	A	2019.08	Triploid rainbow trout	50 ± 3	56
GS2019-4	Gansu	MW662090	A	2019.08	Triploid rainbow trout	4 ± 1	75
GS2020-2 ^d^	Gansu	MW662092	A	2020.08	Triploid rainbow trout	8 ± 2	68
GS2020-1 ^d^	Gansu	MW662091	J	2020.05	Triploid rainbow trout	3 ± 1	80
QH2019-1	Qinghai	MW662093	B	2019.03	Triploid rainbow trout	145 ± 10	35
JL2019-1	Jilin	MW662094	C	2019.03	Triploid rainbow trout	4 ± 1	0
LN2018-1	Liaoning	MW662095	D	2018.10	Triploid rainbow trout	450 ± 25	10
LN2019-1 *	Liaoning	MW662096	D	2019.04	Triploid rainbow trout	50 ± 15	75
LN2019-2	Liaoning	MW662097	D	2019.04	Triploid rainbow trout	50 ± 10	55
XZ2019-1	Tibet	MW662098	F	2019.08	Brown trout	750 ± 45	10
XZ2019-2	Tibet	MW662099	G	2019.08	Brown trout	3.5 ± 1	60
HLJ2019-1	Heilongjiang	MW662100	E	2019.08	Hucho taimen	5 ± 1	15
HLJ2019-2	Heilongjiang	MW662101	E	2019.08	White-spotted char	3.5 ± 1	35
HLJ2019-3	Heilongjiang	MW662102	E	2019.08	Brook trout	5 ± 2	40
HLJ2019-4	Heilongjiang	MW662103	E	2019.08	Brown trout	30 ± 6	35
HLJ2019-5	Heilongjiang	MW662104	E	2019.08	Crucian carp	4 ± 1	NA ^e^
HLJ2019-6	Heilongjiang	MW662105	E	2019.08	Masu salmon	4 ± 2	30
HLJ2019-7	Heilongjiang	MW662106	E	2019.08	Brook trout	50 ± 10	20
HLJ2019-8	Heilongjiang	MW662107	E	2019.08	Brown trout	10 ± 3	60
BJ2020-1 ^d^	Beijing	MW662108	I	2020.05	Rainbow trout	3 ± 1	50
ChRtm213	Yunnan	KX234591	H	2013.05	Triploid rainbow trout	7 ± 2	70
WZ20165	Sichuan	KX355401	/	2016.04	Rainbow trout	Unknown	100

* IPNV strains were co-infected with infectious hematopoietic necrosis virus (IHNV). ^a^ Farm sites are indicated as random letters. ^b^ Latin names of hosts are as follows: Rainbow trout, *Oncorhynchus mykiss*; Brown trout, *Salmo trutta*; Crucian carp, *Carassius auratus*; Masu salmon, *Oncorhynchus masou masou*. White-spotted char, *Salvelinus leucomaenis*; Brook trout, *Salvelinus fontinalis.* The common name of *Hucho taimen* is unknown. ^c^ Approximate natural mortality rate. ^d^ Genogroup V, the rest are genogroup I. ^e^ Dead crucian carp was occasionally found, and the mortality was not available.

**Table 4:** Nucleotide (Amino acid) sequence identity of Chinese IPNV with other Aquabirnavirus.

	Chinese Genogroup I IPNV	Chinese Genogroup V IPNV
(1) genogroup I	90.2–98.5% (97.2–99.4%)	80.6–81.0% (88.4–89.0%)
(2) genogroup II	80.24–87.78% (89.04–89.98%)	84.9–85.7% (90.6–91.0%)
(3) genogroup III	80.54–81.06% (88.24–89.31%)	80.0–88.3% (90.0–92.4%)
(4) genogroup IV	79.94–80.48% (86.65–87.97%)	84.2–85.9% (91.0–91.2%)
(5) genogroup V	77.48–90.76% (84.08–90.96%)	97.1–100% (97.6–100%)
(6) genogroup VI	74.96–76.32% (83.71–85.75%)	77.4–78.7% (88.2–87.8%)
(7) genogroup VII	75.67–85.13% (88.69–90.27%)	83.1–83.6% (90.0–90.2%)

**Table 5:** Virulent signature analysis of Chinese genogroup V IPNV strains.

Amino Acid Site on VP2	Highly Virulent	Moderately Virulent	Avirulent	GS2020-1	BJ2020-2	GS2020-3
199	Thr [8]	Ile [8]	Ile [8]	Ile	Ile	Ile
217	Thr [8,36,38,40]	Ala [36] Pro [8,40]	Pro [8,38]	Pro	Pro	Pro
221	Ala [38,40] Thr [8]	Ala [8,40]	Thr [38,40] Ala [8]	Thr	Thr	Thr
247	Thr [8]	Ala [8]	Ala [8]	Ala	Ala	Ala
286	Lys [36]	Ala [36]	-	Gly	Gly	Gly
288	Val [8]	Ala [8]	Val [8]	Val	Val	Val
500	Tyr [8]	His [8]	His [8]	Tyr	Tyr	Tyr

## Data Availability

The data presented in this study are available in this article and supplementary material here.

## Supplementary Materials

The following are available online at https://www.mdpi.com/1999-4915/13/3/488/s1. Figure S1: date–randomization test (DRT) of genogroup I IPNV strains. Table S1: marginal likelihoods of different combinations of clock model and tree prior, estimators of the marginal likelihood—path sampling (PS). Table S2: marginal likelihoods of different combinations of clock model and tree prior, stepping-stone (SS) sampling.
