# Supplementary material for: Molecular Evolution of Infectious Pancreatic Necrosis Virus in China
Source: Viruses. 2021 Mar 16;13(3):488. doi: 10.3390/v13030488 (PMC7998647; doi:10.3390/v13030488)
Supplement: Supplementary file 1 [file viruses-13-00488-s001.pdf]

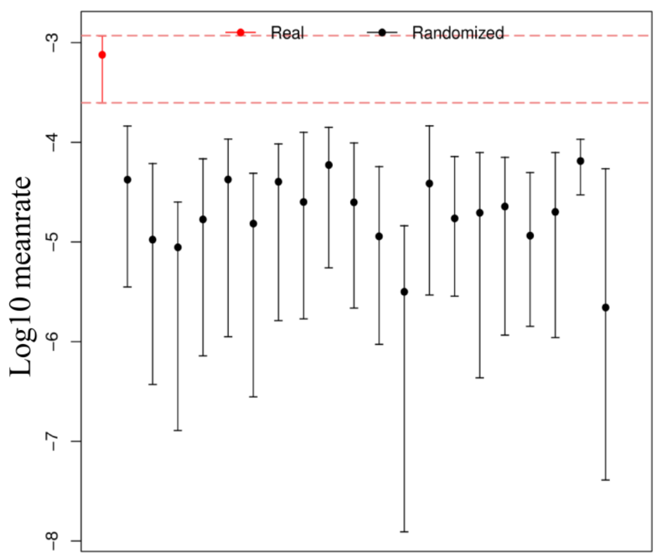

Figure S1. Date-randomization test (DRT) of genogroup 1 IPNV strains.

Table S1 Marginal likelihoods of different combinations of clock model and tree prior, estimators of the marginal likelihood—path sampling (PS)

Molecular clock model	Coalescent tree prior	Log marginal likelihood
Strict clock	Bayesian skyline	-4171.045
Strict clock	Exponential growth	-4189.685
Strict clock	Constant size	-4188.345
***Uncorrelated lognormal relaxed clock**	**Bayesian skyline**	**-4157.578**
Uncorrelated lognormal relaxed clock	Exponential growth	-4174.000
Uncorrelated lognormal relaxed clock	Constant size	-4169.600

*the combination of clock model and tree prior that best fit our sequences data in this study.

Table S2 Marginal likelihoods of different combinations of clock model and tree prior, stepping-stone (SS) sampling

Molecular clock model	Coalescent tree prior	Log marginal likelihood
Strict clock	Bayesian skyline	-4172.473
Strict clock	Exponential growth	-4191.000
Strict clock	Constant size	-4188.846
***Uncorrelated lognormal relaxed clock**	**Bayesian skyline**	**-4158.026**
Uncorrelated lognormal relaxed clock	Exponential growth	-4175.153
Uncorrelated lognormal relaxed clock	Constant size	-4170.961

*the combination of clock model and tree prior that best fit our sequences data in this study.
